# Non-invasive method to detect high respiratory effort and transpulmonary driving pressures in COVID-19 patients during mechanical ventilation

**DOI:** 10.1186/s13613-021-00821-9

**Published:** 2021-02-08

**Authors:** Lisanne Roesthuis, Maarten van den Berg, Hans van der Hoeven

**Affiliations:** Department of Intensive Care Medicine, Radboud University Medical Center, Geert Grooteplein-Zuid 10, 6525 GA Nijmegen, The Netherlands

**Keywords:** Coronavirus disease 2019, Respiratory monitoring, Occlusion pressure, Dynamic transpulmonary pressure, Respiratory muscle pressure, Respiratory effort

## Abstract

**Background:**

High respiratory drive in mechanically ventilated patients with spontaneous breathing effort may cause excessive lung stress and strain and muscle loading. Therefore, it is important to have a reliable estimate of respiratory effort to guarantee lung and diaphragm protective mechanical ventilation. Recently, a novel non-invasive method was found to detect excessive dynamic transpulmonary driving pressure (∆*P*_L_) and respiratory muscle pressure (*P*_mus_) with reasonable accuracy. During the Coronavirus disease 2019 (COVID-19) pandemic, it was impossible to obtain the gold standard for respiratory effort, esophageal manometry, in every patient. Therefore, we investigated whether this novel non-invasive method could also be applied in COVID-19 patients.

**Methods:**

∆*P*_L_ and *P*_mus_ were derived from esophageal manometry in COVID-19 patients. In addition, ∆*P*_L_ and *P*_mus_ were computed from the occlusion pressure (∆*P*_occ_) obtained during an expiratory occlusion maneuver. Measured and computed ∆*P*_L_ and *P*_mus_ were compared and discriminative performance for excessive ∆*P*_L_ and *P*_mus_ was assessed. The relation between occlusion pressure and respiratory effort was also assessed.

**Results:**

Thirteen patients were included. Patients had a low dynamic lung compliance [24 (20–31) mL/cmH_2_O], high ∆*P*_L_ (25 ± 6 cmH_2_O) and high *P*_mus_ (16 ± 7 cmH_2_O). Low agreement was found between measured and computed ∆*P*_L_ and *P*_mus_. Excessive ∆*P*_L_ > 20 cmH_2_O and *P*_mus_ > 15 cmH_2_O were accurately detected (area under the receiver operating curve (AUROC) 1.00 [95% confidence interval (CI), 1.00–1.00], sensitivity 100% (95% CI, 72–100%) and specificity 100% (95% CI, 16–100%) and AUROC 0.98 (95% CI, 0.90–1.00), sensitivity 100% (95% CI, 54–100%) and specificity 86% (95% CI, 42–100%), respectively). Respiratory effort calculated per minute was highly correlated with ∆*P*_occ_ (for esophageal pressure time product per minute (PTP_es/min_) *r*^2^ = 0.73; *P* = 0.0002 and work of breathing (WOB) *r*^2^ = 0.85; *P* < 0.0001).

**Conclusions:**

∆*P*_L_ and *P*_mus_ can be computed from an expiratory occlusion maneuver and can predict excessive ∆*P*_L_ and *P*_mus_ in patients with COVID-19 with high accuracy.

## Background

Maintaining spontaneous breathing effort in mechanically ventilated patients limits respiratory muscle disuse and atrophy [1–4]. Too high respiratory effort may lead to excessive lung stress and strain causing lung injury on one hand. On the other hand, it may lead to excessive muscle loading causing muscle injury (mainly diaphragm injury) leading to muscle dysfunction [5]. High respiratory drive and effort frequently exist in critically ill patients, mainly due to insufficient ventilator assistance and sedation, but evidence also suggests biological predisposition (e.g., pulmonary and systemic inflammation, lung mechanical heterogeneity) plays a role as well. Therefore, it is important to have a reliable estimate of respiratory effort to enable lung and diaphragm protective mechanical ventilation [6–8].

The gold standard to obtain respiratory effort is esophageal manometry. This technique is minimally invasive, requires appropriate equipment and expertise, and can be time consuming. Other monitoring techniques or parameters only reflect respiratory drive (*P*_0.1_ and electrical activity of the diaphragm) or muscle loading (diaphragm ultrasound) and provide only limited information about lung stress and strain (plateau pressure and driving pressure) [7]. Recently, Bertoni et al. [9] demonstrated that dynamic transpulmonary driving pressure (∆*P*_L_) and respiratory muscle pressure (*P*_mus_) can be estimated from the maximal decline in airway pressure (*P*_aw_) from positive end-expiratory pressure (PEEP) during an expiratory occlusion maneuver (∆*P*_occ_). Direct estimates of ∆*P*_L_ and *P*_mus_ were unreliable, excessive ∆*P*_L_ and *P*_mus_, however, could be predicted with reasonable accuracy.

Coronavirus disease 2019 (COVID-19) is a new type of lung disease [10–12] originating from Wuhan, China, in December 2019. Because of the sheer number of mechanically ventilated patients with severe lung disease, it was impossible to measure esophageal pressure to assess respiratory mechanics in every patient. Therefore, we estimated ∆*P*_L_ and *P*_mus_ according to Bertoni et al. [9] in every COVID-19 patient with spontaneous breathing effort as part of standard patient care. If computed ∆*P*_L_ and/or *P*_mus_ were excessive (i.e., higher than *P*_mus_ 13–15 cmH_2_O and ∆*P*_L_ 16–17 cmH_2_O), or if patients received prolonged mechanical ventilation with no progress (i.e., ≥14 days) or if patients remained hypercapnic (PaCO_2_ ≥ 60 mmHg), respiratory mechanics was assessed by esophageal manometry for clinical purposes.

The aim of this paper is to describe respiratory mechanics in mechanically ventilated COVID-19 patients with spontaneous breathing effort, to compute ∆*P*_L_ and *P*_mus_ from ∆*P*_occ_ and assess the discriminative performance for excessive ∆*P*_L_ and *P*_mus_, and to assess the relation between ∆*P*_occ_ and respiratory effort.

## Methods

### Study population

Dynamic transpulmonary driving pressure and respiratory muscle pressure were assessed in COVID-19 patients admitted to the Intensive Care Unit of the Radboud University Medical Center according to Bertoni et al. [9] as follows:

1. computed ∆*P*_L_ = (peak *P*_aw_ − PEEP)—2/3 × ∆*P*_occ_.
2. computed *P*_mus_ = − 3/4 × ∆*P*_occ_.

If patients had high respiratory effort and/or high dynamic transpulmonary driving pressure (i.e., computed *P*_mus_ 13–15 cmH_2_O and ∆*P*_L_ 16–17 cmH_2_O or higher), prolonged mechanical ventilation without clinical progress (i.e.,  ≥ 14 days) or remained hypercapnic (PaCO_2_ ≥ 60 mmHg), esophageal manometry was obtained as part of our standard clinical protocol. Patients or their legal representatives were informed about the measurements.

### Study protocol

This was an observational study. All patients were ventilated with a Servo-i/u ventilator (Getinge, Sölna, Sweden). Ventilator settings were set by the treating intensivist. Patients received a nasogastric catheter with esophageal balloon [Cooper (Cooper Surgical, Trumbull, USA) or Neurovent (NeuroVent Research Inc, Toronto, Canada)] to obtain esophageal pressure (*P*_es_). Catheter position was validated using the dynamic occlusion test [13]. A total of 3–4 manual expiratory occlusions (lasting ~ 1–2 s) were performed during a 10–15 min recording per patient. After the recordings, ventilator settings or sedation strategies were adjusted, if deemed necessary, in accordance with the treating intensivist. Being an observational study, the effect of different ventilator settings or sedatives was not investigated.

### Data acquisition

Ventilator flow and airway pressure (*P*_aw_) were obtained (sample frequency 100 Hz) by connecting a RS-232 cable via the serial port of the Servo-i/u to a dedicated measurement set-up using Servotracker software (Servotracker release 4.2, Getinge, Sölna, Sweden). The esophageal balloon (i.e., *P*_es_) and a T-piece connected to the expiration port of Servo-i/u (i.e., *P*_aw_) were coupled to pressure transducers and acquired (sample frequency 100 Hz) using a dedicated measurement set-up (Biopac MP160, BIOPAC Inc., USA). Signals were synchronized offline based on *P*_aw_ tracings that were acquired using both software programs. Brief manual expiratory occlusions (lasting ~ 1–2 s) were performed to enable offline synchronization. Data were processed and analyzed offline using Matlab R2018a (Mathworks, Natick, MA, USA).

### Signal analysis

**Fig. 1:**
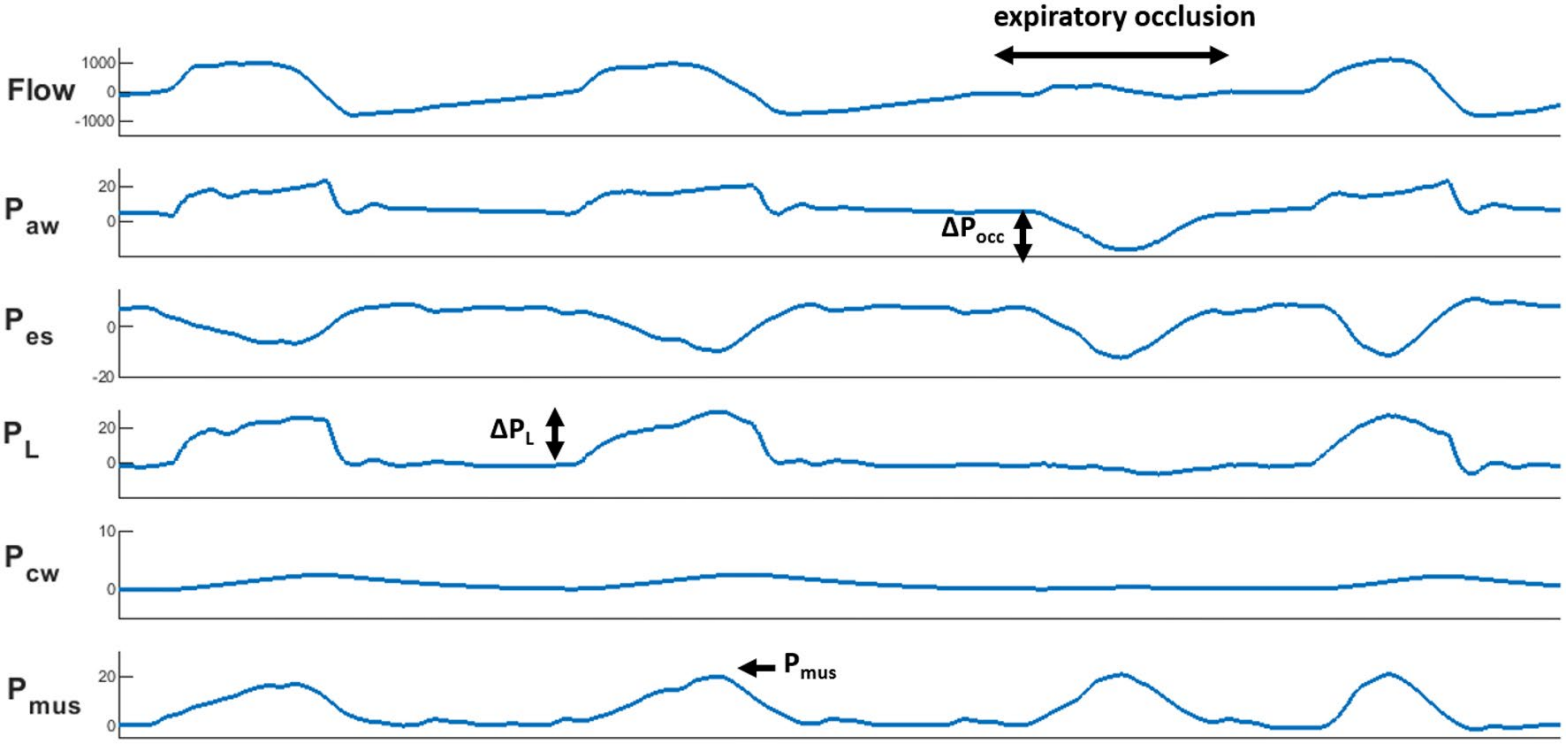
Flow and pressure tracings showing an expiratory occlusion maneuver. From top to bottom: flow (in mL/s), airway pressure (*P*_aw_), esophageal pressure (*P*_es_), transpulmonary pressure (*P*_L_) (*P*_aw_ – *P*_es_), chest wall elastic recoil pressure (*P*_cw_) (tidal volume × estimated chest wall elastance) and respiratory muscle pressure (*P*_mus_) (*P*_cw_ – P_es_) (pressures in cmH_2_O). During an expiratory occlusion maneuver the patient inhales against a closed valve, resulting in a decrease in airway pressure. The maximal deflection in *P*_aw_ from positive end-expiratory pressure is defined as occlusion pressure (∆*P*_occ_). From this ∆*P*_L_ and *P*_mus_ were computed and compared with true dynamic lung stress (increase in *P*_L_ from onset to peak during inspiration) and true respiratory effort (peak *P*_mus_ during inspiration)

The occlusion pressure (∆*P*_occ_) was defined as the maximal deflection in *P*_aw_ from positive end-expiratory pressure (PEEP) during an expiratory occlusion maneuver (Fig. 1). The decrease in *P*_es_ during the first 100 ms of this maneuver was computed as *P*_0.1_. Transpulmonary pressure (*P*_L_) was determined by subtracting *P*_es_ from *P*_aw_. Dynamic transpulmonary driving pressure (∆*P*_L_) was computed from onset to peak during inspiration. Dynamic lung compliance (*C*_dyn_) was calculated as tidal volume divided by the increase in *P*_L_ between points of zero flow. Chest wall elastance (*E*_cw_) was estimated based on predicted vital capacity [9, 14], from this chest wall elastic recoil pressure (*P*_cw_) was computed as the product of tidal volume and *E*_cw_. The pressure generated by the respiratory muscles (*P*_mus_) was calculated as *P*_cw_ minus *P*_es_. The integral of the product of *P*_mus_ and tidal volume represents work of breathing (WOB), calculated per liter and per minute. The integral of *P*_mus_ over time is defined as esophageal pressure–time product (PTP_es_), calculated per breath and per minute [14, 15].

Data obtained during expiratory occlusion maneuvers were averaged. Data were analyzed on a breath-by-breath basis and averaged over at least a 4-min period free of artifacts or esophageal contractions. Only recordings where ∆*P*_es_/∆*P*_occ_ was between 0.8 and 1.2 were included in the analysis.

### Statistical analysis

Normality was tested and data are presented accordingly as mean ± standard deviation (SD) or as median [interquartile range (IQR)]. Measured and computed ∆*P*_L_ and *P*_mus_ were compared using Bland–Altman analysis. Receiver operating characteristic curve analysis was performed and sensitivity and specificity were computed to assess the accuracy of computed ∆*P*_L_ and *P*_mus_ to detect excessive ∆*P*_L_ > 20 cmH_2_O and *P*_mus_ > 10 and > 15 cmH_2_O. Linear regression analysis was performed to assess the relationship between ∆*P*_occ_ and respiratory effort. For all tests a two-tailed *P* < 0.05 was considered statistically significant. Statistical analyses were performed with Prism 5 (Graphad software, San Diego, USA).

## Results

### Patient characteristics

Esophageal manometry was obtained in 15 COVID-19 patients between April and July 2020. Two patients were excluded from analysis due to incorrect ∆*P*_es_/∆*P*_occ_. Patient characteristics at time of measurement are shown in Table 1. In general, patients were 61 ± 9 years old, had high PaCO_2_ (63 ± 17 mmHg) and received prolonged mechanical ventilation (41 ± 32 days). Respiratory failure was the main problem.

**Table 1:** Patient characteristics

Subject	Age	Gender	Main medical history	Position	PaO_2_/FiO_2_ ratio	pH	PaCO_2_ (mmHg)	RASS	Days of MV on study day
1	62	M	–	P	175	7.26	74	− 5	17
2	71	M	Asthma, ABPA	S	216	7.43	59	− 4	4
3	69	M	2 × PCI	S	116	7.25	94	− 4	22
4	73	M	COPD Gold II	S	262	7.22	64	− 3	38
5	51	M	Waldeström disease	S	156	7.36	57	− 4	14
6	49	M	–	P	78	7.42	47	− 5	6
7	66	M	OSAS, asthma	S	182	7.47	40	+ 1	42
8	63	M	Hypertension, obesity	S	118	7.31	97	− 4	46
9	53	M	Hodgkin	P	168	7.42	47	− 4	21
10	47	F	Hypertension	S	336	7.42	47	0	66
11	62	M	–	s-L	251	7.41	63	0	80
12	69	M	CABG	S	155	7.38	74	0	109
13	63	M	OSAS, hypertension	S	148	7.33	62	− 1	74

*ABPA* allergic bronchopulmonary aspergillosis, *AKI* acute kidney injury, *CABG* coronary artery bypass grafting, *COPD* chronic obstructive pulmonary disease, *OSAS* obstructive sleep apnea syndrome, *FiO*_*2*_ fraction of inspired oxygen, *MV* mechanical ventilation, *P* prone, *PaCO*_*2*_ partial pressure of carbon dioxide in arterial blood, *PaO*_*2*_ partial pressure of oxygen in arterial blood, *PCI* percutaneous coronary intervention, *S* supine, *s-L* semi-lateral due to decubitus

Respiratory parameters are shown in Table 2. Only in patient 7 it was not possible to analyze a 4-min period due to continuous esophageal contractions. Patients had a low *C*_dyn_ [24 (20–31) mL/cmH_2_O], high ∆*P*_L_ (25 ± 6 cmH_2_O) and high *P*_mus_ (16 ± 7 cmH_2_O).

**Table 2:** Respiratory parameters

Subject	PS (cmH_2_O)	PEEP (cmH_2_O)	RR (#/min)	Vt/IBW (mL)	∆*P*_occ_ (cmH_2_O)	*P*_0.1_ (cmH_2_O)	*C*_dyn_ (mL/cmH_2_O)	∆*P*_L_ (cmH_2_O)	∆*P*_L, computed_ (cmH_2_O)	*P*_mus_ (cmH_2_O)	*P*_mus, computed_ (cmH_2_O)	WOB (J/L)	WOB (J/min)	PTP_es breath_ (cmH_2_O*s)	PTP_es/min_ (cmH_2_O × s × min^−1^)
1	20	8	45	5.0	7	4.5	21	23	26	9	5	0.6	9.8	1.9	85
2	14	7	20	7.8	24	6.2	26	30	31	20	18	1.5	17.9	8.6	176
3	20	12	23	5.8	13	2.3	19	24	29	8	9	0.3	2.6	1.6	38
4	14	10	28	6.1	6	2.5	40	20	19	9	4	0.6	7.5	3.0	83
5	8	10	30	6.0	25	5.4	31	29	27	25	19	1.7	25.9	8.0	239
6	5	11	24	6.2	21	2.3	32	23	22	20	16	1.4	16.4	9.4	230
7	8	8	41	7.7	45	6.6	19	33	40	29	34	1.7	37.5	9.4	381
8	16	10	29	5.5	18	6.0	27	29	31	16	13	1.2	13.5	4.6	134
9	16	8	28	6.2	18	2.5	20	29	31	15	13	0.7	8.6	4.4	125
10	2	6	33	5.1	9	2.2	51	11	11	9	6	0.7	6.5	3.8	126
11	13	5	35	6.0	22	5.2	21	29	32	17	16	1.3	19.2	7.8	271
12	10	5	28	5.1	12	3.5	24	21	20	12	9	0.7	7.9	6.0	167
13	12	10	24	5.5	11	1.9	20	24	20	13	8	0.8	7.9	7.8	188

*C*_*dyn*_ dynamic lung compliance, *IBW* ideal body weight, *P*_*0.1*_ decline in esophageal pressure during the first 100 ms of an expiratory occlusion maneuver, *PEEP* positive end-expiratory pressure, *∆P*_*L*_ dynamic transpulmonary driving pressure, *P*_*mus*_ respiratory muscle pressure, *∆P*_*occ*_ occlusion pressure, *PS* pressure support, *PTP*_*es*_ pressure time product of esophageal pressure, *RR* respiratory rate, *WOB* work of breathing

### Computed ∆*P*_L_ and *P*_mus_

**Fig. 2:**
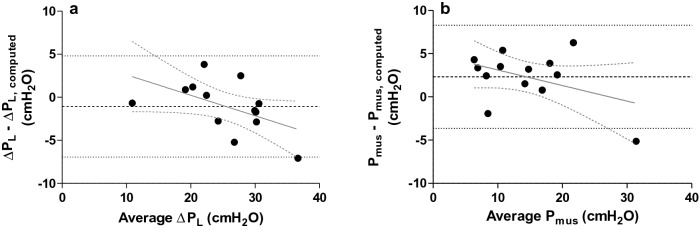
Bland–Altman plots with regression lines in which measured and computed dynamic transpulmonary driving pressure (∆*P*_L_) (**a**) and respiratory muscle pressure (*P*_mus_) (**b**) are compared. Computed ∆P_L_ overestimates measured ∆*P*_L_ (**a**) (− 1.1 ± 5.9 cmH_2_O (bias ± 95% limits of agreement), while computed *P*_mus_ underestimates measured *P*_mus_ (**b**) (2.3 ± 6.0 cmH_2_O). Limits of agreement are large for both parameters. There was no significant trend in differences (for ∆*P*_L_ *r*^2^ = 0.27; *P* = 0.06 and for *P*_mus_ *r*^2^ = 0.18; *P* = 0.15)

Bland–Altman analysis showed low bias, but wide limits of agreement between measured and computed ∆*P*_L_ [− 1.1 ± 5.9 cmH_2_O (bias ± 95% limits of agreement)] (Fig. 2a). Bias between measured and computed *P*_mus_ was higher and limits of agreement were equally wide (2.3 ± 6.0 cmH_2_O) (Fig. 2b). This means there is poor agreement between measured and computed ∆*P*_L_ and *P*_mus_.

**Fig. 3:**
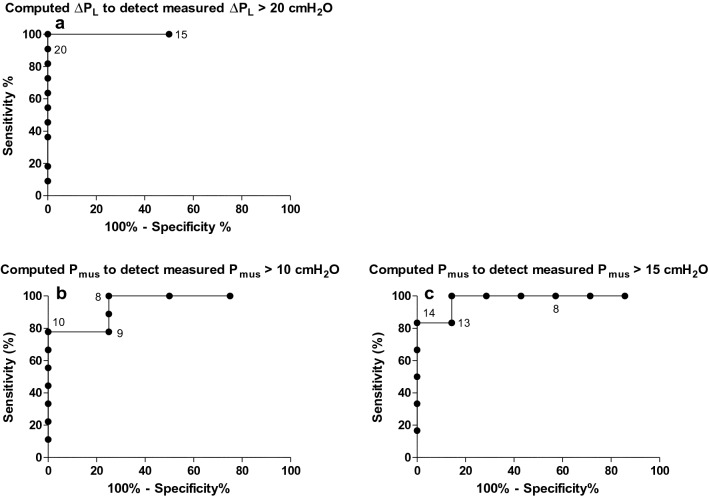
Receiver operating characteristic curves (ROC) showing the discriminative performance of computed transpulmonary driving pressure (∆*P*_L_) to detect measured excessive (> 20 cmH_2_O) ∆*P*_L_ [area under the ROC (AUROC) 1.00 (95% confidence interval (CI), 1.00–1.00)] (**a**) and computed respiratory muscle pressure (*P*_mus_) to detect measured *P*_mus_ > 10 cmH_2_O [AUROC 0.94 (95% CI, 0.81–1.00)] (**b**) and 15 cmH_2_O (AUROC 0.98 (95% CI, 0.90–1.00)] (**c**). Threshold values are shown as points on the curves

Receiver operating characteristic curve analysis was performed to assess the discriminative performance to predict excessive dynamic lung stress and respiratory effort (Fig. 3; Table 3). Excessive ∆*P*_L_ > 20 cmH_2_O was accurately predicted by computed ∆*P*_L_ > 19 cmH_2_O [with area under the receiver operating curve (AUROC) 1.00 (95% confidence interval (CI), 1.00–1.00), sensitivity 100% (95% CI, 72–100%) and specificity 100% (95% CI, 16–100%)]. Discriminative performance for *P*_mus_ > 10 cmH_2_O was only moderate, but was acceptable for *P*_mus_ > 15 cmH_2_O with computed *P*_mus_ > 13 cmH_2_O [with AUROC 0.98 (95% CI, 0.90–1.00), sensitivity 100% (95% CI, 54–100%) and specificity 86% (95% CI, 42–100%)] (Fig. 3).

**Table 3:** Discriminative performance

Parameter	Threshold measured value	Threshold computed value for excessive value	Area under receiver operating characteristic curve (95% CI)	Sensitivity (95% CI)	Specificity (95% CI)
Excessive dynamic lung stress	∆*P*_L_ > 20 cmH_2_O	Computed ∆*P*_L_ > 18 cmH_2_O	1.00 (1.00–1.00)	100% (72–100%)	50% (1–99%)
		Computed ∆*P*_L_ > 19 cmH_2_O		100% (72–100%)	100% (16–100%)
		Computed ∆*P*_L_ > 20 cmH_2_O		91% (59–100%)	100% (16–100%)
Excessive respiratory effort	*P*_mus_ > 10 cmH_2_O	Computed *P*_mus_ > 8 cmH_2_O	0.94 (0.81–1.00)	100% (66–100%)	75% (19–99%)
		Computed *P*_mus_ > 9 cmH_2_O		78% (40–100%)	75% (19–99%)
		Computed *P*_mus_ > 10 cmH_2_O		78% (40–97%)	100% (40–100%)
	*P*_mus_ > 15 cmH_2_O	Computed *P*_mus_ > 13 cmH_2_O	0.98 (0.90–1.00)	100% (54–100%)	86% (42–100%)
		Computed *P*_mus_ > 14 cmH_2_O		83% (36–100%)	100% (59–100%)
		Computed *P*_mus_ > 15 cmH_2_O		83% (36–100%)	100% (59–100%)

*∆P*_*L*_ dynamic transpulmonary driving pressure, *P*_*mus*_ respiratory muscle pressure

### ∆*P*_occ_ and respiratory effort

**Fig. 4:**
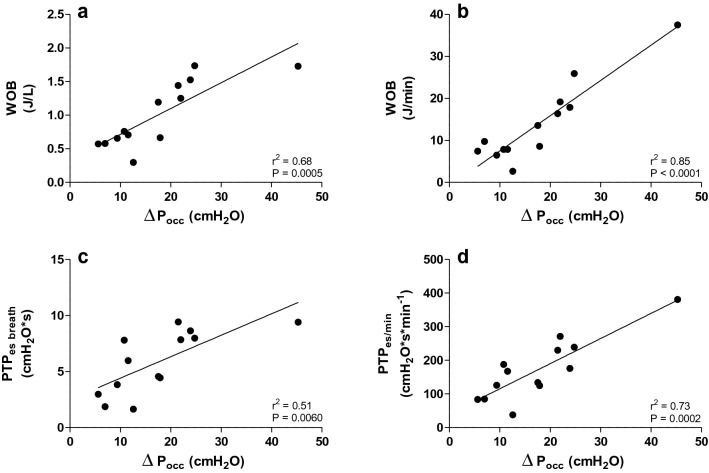
Relation between occlusion pressure (∆*P*_occ_) and respiratory effort. ∆*P*_occ_ is moderately correlated with work of breathing (WOB) calculated per liter (**a**) and pressure time product of esophageal pressure per breath (PTP_es breath_) (**c**). Higher correlations were found between ∆*P*_occ_ and respiratory effort when respiratory effort was calculated per minute (**b**, **d**)

∆*P*_occ_ was correlated with respiratory effort (Fig. 4). Only moderate correlations were found between ∆*P*_occ_ and PTP_es breath_ (*r*^2^ = 0.51; *P* = 0.0060) and WOB (calculated per liter) (*r*^2^ = 0.68; *P* = 0.0005). Respiratory effort calculated per minute showed much better correlations with ∆*P*_occ_ (for PTP_es/min_ *r*^2^ = 0.73; *P* = 0.0002 and WOB *r*^2^ = 0.85; *P* < 0.0001).

## Discussion

We demonstrate that the mechanically ventilated COVID-19 patients with spontaneous breathing effort included in this study received prolonged mechanical ventilation, had a low dynamic lung compliance, high dynamic transpulmonary driving pressures and high respiratory effort. Dynamic transpulmonary driving pressure and respiratory muscle pressure were estimated from the maximal decline in airway pressure from PEEP during an expiratory occlusion maneuver. Computed ∆*P*_L_ and *P*_mus_ are unreliable for direct estimates of ∆*P*_L_ and *P*_mus_ derived from esophageal manometry, as analysis showed poor agreement between computed and measured values. However, they can predict excessive ∆*P*_L_ (> 20 cmH_2_O) and *P*_mus_ (> 15 cmH_2_O) with high sensitivity and specificity. The occlusion pressure is highly correlated with respiratory effort per minute.

### Dynamic lung stress and respiratory effort

Maintaining spontaneous breathing effort during mechanical ventilation has become increasingly important in recent years, due to accumulating evidence for over-assistance myotrauma not only during controlled mechanical ventilation, but also during high levels of pressure support ventilation [1–5]. Too high respiratory effort, however, can also cause lung and/or diaphragm injury. This might not be that obvious when relying only on plateau and driving pressures on the ventilator screen. The pressure generated by the respiratory muscles (i.e., *P*_mus_) might in fact be quite high and thus the pleural pressure (i.e., *P*_es_) quite negative, despite high levels of pressure support. Indirect evidence suggests that high *P*_mus_ may cause load-induced muscle injury and dysfunction [5, 6]. Negative pleural pressures in an already injured lung increase transpulmonary pressures and thus lung stress and strain and worsen vascular leakage [i.e., patient-self inflicted lung injury (P-SILI)] [16]. In our study, patients had a relatively high *P*_mus_ and PaCO_2_. Apparently, they were not able to increase *P*_mus_ to achieve a normal PaCO_2_. Patients had a high respiratory frequency, but this was insufficient in most patients to meet ventilatory demands as they had high dead space ventilation reflecting severe gas exchange disorders (Additional file 1: Table S1) [17]. ∆*P*_occ_ was only moderately correlated with PTP_es breath_ and WOB (J/L), but highly correlated when respiratory effort was multiplied with respiratory frequency [i.e., PTP_es/min_ and WOB (J/min)]. Telias et al. [18] observed something similar for *P*_0.1_, which correlated better with respiratory effort per minute as compared to respiratory effort per breath. Together, the data from our study and the study of Telias et al. [18] suggest that in response to high respiratory drive critically ill patients increase respiratory frequency rather than tidal volume, probably due to a combination of respiratory muscle weakness and decreased lung compliance, limiting the ability to increase effort per breath [7, 19, 20].

### Clinical implications

Bertoni et al. [9] provided a novel non-invasive method to compute ∆*P*_L_ and *P*_mus_ from ∆*P*_occ_ in mechanically ventilated patients with spontaneous breathing effort. We demonstrated that this novel method can also be applied in COVID-19 patients. In accordance with Bertoni et al. [9], computed ∆*P*_L_ and *P*_mus_ cannot directly replace ∆*P*_L_ and *P*_mus_ derived from esophageal manometry. In the external validation cohort they found reasonable discriminative performance for ∆*P*_L_ > 15 cmH_2_O and *P*_mus_ > 10 cmH_2_O. In this study, we were able to show that computed values can also be used to predict excessive ∆*P*_L_ (> 20 cmH_2_O) and *P*_mus_ (> 15 cmH_2_O). This is very useful when it is not feasible to perform esophageal manometry for various reasons.

COVID-19 patients have severely injured lungs and are prone to high respiratory effort, necessitating close monitoring to enable lung and diaphragm protective ventilation [6, 8]. If computed ∆*P*_L_ and/or *P*_mus_ are/is excessively high, one can decide to measure esophageal pressures. If that is not feasible, ventilator settings should be changed followed by appropriate sedation to keep computed ∆*P*_L_ and *P*_mus_ within the clinically acceptable range based on most recent studies and reviews [6, 8, 9]. Excessive sedation, however, can lead to insufficient respiratory effort (i.e., diminished ∆*P*_occ_) and increased patient ventilator asynchronies [8].

### Limitations

This study has some limitations. First, the relatively small sample size. However, many physiological studies with critically ill patients are limited in sample size. For example, the external validation cohort in the study by Bertoni et al. [9] only included 12 patients. Second, there is a selection bias. Only patients with computed high respiratory effort and/or high dynamic transpulmonary driving pressure, prolonged mechanical ventilation and/or who were hypercapnic, were included in the study. Therefore, we found relatively high measured ∆*P*_L_ and *P*_mus_. Third, limitations in measured ∆*P*_L_ and *P*_mus_. ∆*P*_L_ is the dynamic transpulmonary driving pressure, therefore it may overestimate lung stress due to the resistance component. Some studies suggest to perform an end-inspiratory occlusion maneuver in the presence of spontaneous breathing activity to obtain semi-static pressure measurements [21, 22]. For *P*_mus_ calculations the chest wall elastance was estimated based on predicted vital capacity. Bertoni et al. [9] demonstrated that predicted values approximated measured values of chest wall elastance.

## Conclusions

In mechanically ventilated COVID-19 patients with spontaneous breathing effort ∆*P*_L_ and *P*_mus_ can be computed from an expiratory occlusion maneuver. Computed ∆*P*_L_ and *P*_mus_ cannot replace ∆*P*_L_ and *P*_mus_ derived from esophageal manometry, but they can predict excessive ∆*P*_L_ and *P*_mus_ with high accuracy. The occlusion pressure is highly correlated with respiratory effort per minute.

## Supplementary Information

**Additional file 1**: **Table S1** Dead space ventilation.

## Data Availability

The datasets used and analyzed during the current study are available from the corresponding author on reasonable request.

## Abbreviations

ABPA: Allergic bronchopulmonary aspergillosis
AUROC: Area under the receiver operating characteristic curve
CABG: Coronary artery bypass grafting
*C*_dyn_: Dynamic lung compliance
CI: Confidence interval
COPD: Chronic obstructive pulmonary disease
COVID-19: Coronavirus disease 2019
*E*_cw_: Chest wall elastance
FiO_2_: Fraction of inspired oxygen
IQR: Interquartile range
OSAS: Obstructive sleep apnea syndrome
MV: Mechanical ventilation
*P*_0.1_: Decline in esophageal pressure during the first 100 ms of an expiratory occlusion maneuver
PaCO_2_: Partial pressure of carbon dioxide in arterial blood
PaO_2_: Partial pressure of oxygen in arterial blood
*P*_aw_: Airway pressure
PCI: Percutaneous coronary intervention
*P*_cw_: Chest wall elastic recoil pressure
PEEP: Positive end-expiratory pressure
*P*_es_: Esophageal pressure
(∆)*P*_L_: (Dynamic) transpulmonary driving pressure
*P*_mus_: Respiratory muscle pressure
*P*_occ_: Occlusion pressure
PS: Pressure support
PTP_es_: Pressure time product of esophageal pressure
RR: Respiratory rate
SD: Standard deviation
WOB: Work of breathing
