# Supplementary material for: Non-invasive method to detect high respiratory effort and transpulmonary driving pressures in COVID-19 patients during mechanical ventilation
Source: Ann Intensive Care. 2021 Feb 8;11:26. doi: 10.1186/s13613-021-00821-9 (PMC7868882; doi:10.1186/s13613-021-00821-9)
Supplement: Supplementary file 1 — Additional file 1: Table S1 Dead space ventilation. [file 13613_2021_821_MOESM1_ESM.docx]

**Table S1 Dead space ventilation**

| **Subject** | **Bohr (%)** | **Enghoff (%)** |
| --- | --- | --- |
| 7 | 64 | 74 |
| 8 | 69 | 80 |
| 9 | 64 | 67 |
| 10 | 54 | 55 |
| 11 | - | 76 |
| 12 | - | 73 |
| 13 | 63 | 76 |

**-** Bohr could not be determined due to steep phase III of the volumetric capnogram due to severe heterogeneity of the lung
